# Necrotizing Sarcoid Granulomatosis with Pneumothorax

**DOI:** 10.1155/2019/4648720

**Published:** 2019-05-30

**Authors:** Mihan Pourabdollah, Seyed Reza Saghebi, Mitra Abdolahi, Mitra Sadat Rezaei

**Affiliations:** ^1^Pediatric Respiratory Diseases Research Center, National Research Institute of Tuberculosis and Lung Diseases, Shahid Beheshti University of Medical Sciences, Tehran, Iran; ^2^Thoracic Surgery Research Center, National Research Institute of Tuberculosis and Lung Diseases, Shahid Beheshti University of Medical Sciences, Tehran, Iran; ^3^Department of Pathology, School of Medicine, Shahid Beheshti University of Medical Sciences, Tehran, Iran; ^4^Virology Research Center, National Research Institute of Tuberculosis and Lung Diseases, Shahid Beheshti University of Medical Sciences, Tehran, Iran

## Abstract

Necrotizing sarcoid granulomatosis (NSG) is a rare granulomatous pneumonitis which is composed of a background of sarcoidosis-like granulomas, granulomatous vasculitis, and variable amount of necrosis. We reported a case of a 38-year-old nonsmoking woman presented with left-sided chest pain and dyspnea for three days. Chest CT scan exhibited collapse consolidation of the left lower lobe with the presence of two separated small-sized cystic lesions within the collapsed segment. Lung lesion resection was performed, and histological examination confirmed the diagnosis by excluding other causes of granulomatous diseases. The prognosis of NSG is favorable, and medical treatment is usually not necessary, as well as in our case. NSG is a rare disease with nonspecific symptoms and good prognosis which is frequently confused with Wegener's granulomatosis, sarcoidosis, and Churg–Strauss syndrome. This entity should also be considered as differential diagnosis of necrotizing granulomatous diseases.

## 1. Introduction

Necrotizing sarcoid granulomatosis (NSG) has been distinctly recognized as an infrequent disease. Eloquently, this disease was principally described by Liebow, who defined the pathological features, such as noncaveating epithelial cell granuloma and granulomatous vasculitis with an intermittent degree of necrosis [1]. NSG is typically diagnosed from the pathological features [2]. Based on the evidences, some patients present with asymptomatic condition; nonetheless, the most notable clinical symptoms have been promptly anticipated as fever, fatigue, chest pain, dyspnea, and dry cough [3]. The radiographic presentation consists of multiple bilateral nodules or nodular densities with or without cavitation [4]. Particularly, the disease has confined solely to the lungs. As a matter of fact, the prognosis is quite practical and the recovery without treatment or corticosteroid therapy is occurring [5].

## 2. Case Presentation

A 38-year-old nonsmoking female presented with left-sided chest pain and dyspnea for three consecutive days. The patient was admitted to Masih Daneshvari Hospital on September 19, 2016. She had no history of fever, weight loss, and arthralgia or skin rash. Her blood pressure was 110/70 mmHg, pulse rate was 85, temperature was 37.5°C, and respiratory rate was 28. The breath sound was decreased in the left lower zone of the lung, and other physical examinations were unremarkable.

**Figure 1:**
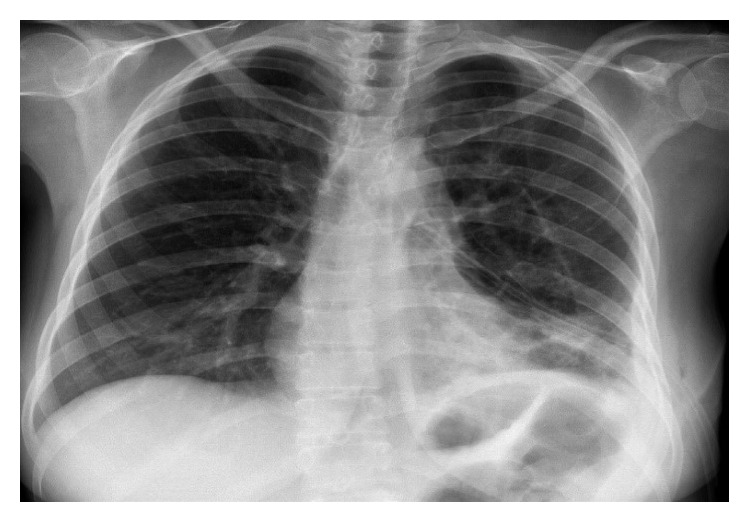
Two pulmonary nodules and basal atelectasis in the left lower lobe with pneumothorax.

**Figure 2:**
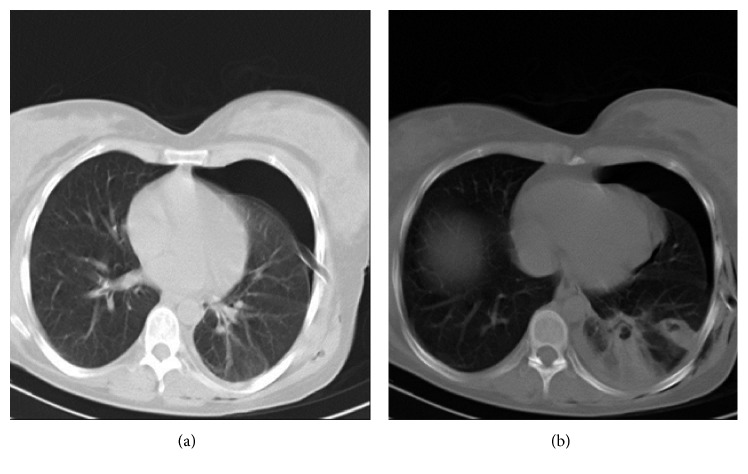
Collapsed consolidation of the left lower lobe with pneumothorax and the presence of relatively two separated small-sized cystic lesions within the collapsed segment.

Chest radiograph manifested two pulmonary nodules and basal atelectasis in the left lower lobe with pneumothorax (Figure 1). Chest CT scan exhibited collapsed consolidation of the left lower lobe with pneumothorax and the presence of relatively two separated small-sized cystic lesions within the collapsed segment (Figure 2).

The laboratory tests revealed the increase in WBC count with predominantly neutrophils; the erythrocyte sedimentation rate and C-reactive protein were elevated. The nitroblue tetrazolium (NTB) blood test was at the 99% accuracy level. Anti-nuclear antibodies (ANAs), anti-neutrophil cytoplasmic antibodies (C-ANCA and P-ANCA), and anti-double-stranded DNA (anti-dsDNA) were negative; in addition, the anti-HIV antibody test was negative. Transparently, the pulmonary function tests showed moderately restriction.

**Figure 3:**
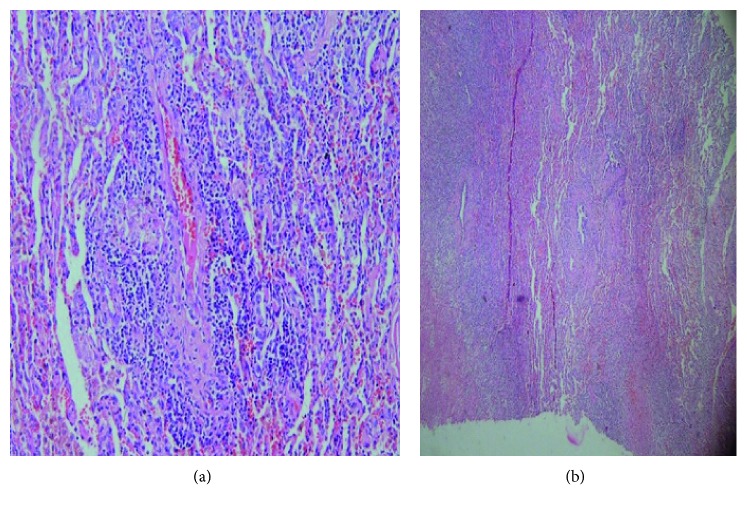
Necrotizing sarcoid granulomatosis vasculitis; the inflammatory infiltrate is confined to the artery wall.

**Figure 4:**
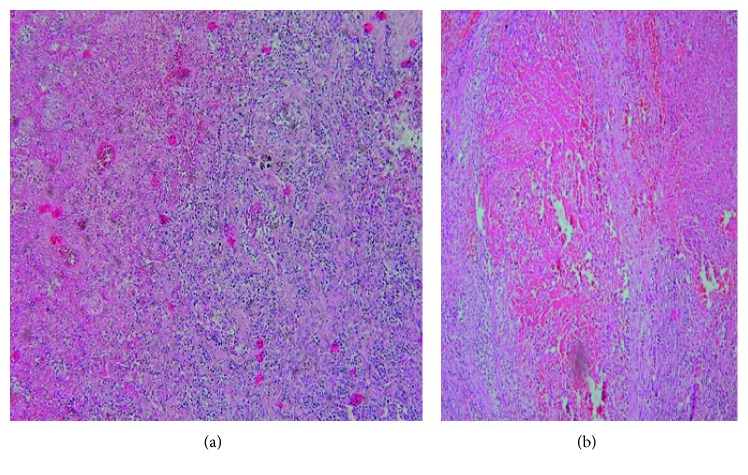
Nonnecrotizing granuloma in conjunction with a large area of necrosis.

Lung lesion resection for two intraparenchymal pulmonary nodules and cavitary lung lesion in the left lower lobe was performed; furthermore, the histological examination showed some epithelioid and giant cell granulomas with necrotizing arteritis in conjunction of a large area of necrosis (Figures 3 and 4). Acid-fast staining, periodic acid-Schiff (PAS) staining, and Gomori methenamine-silver (GMS) staining were negative; therefore, the ultimate diagnosis was necrotizing sarcoid granulomatosis. The prognosis of NSG is favorable, and medical treatment is usually not required. As a matter of fact, in our case report, the patient recovery conspicuously occurred; nevertheless, in some cases, treatment with corticosteroids is quite essential.

## 3. Conclusion

NSG is a rare disease with nonspecific symptoms; however, the prognosis is persuading which affects female more than male with a mean age of approximately 35 years. Morphologically, there are three criteria for NSG composed of sarcoidosis-like granulomas, variable amount of necrosis, and granulomatous vasculitis [6]. NSG etiology and pathogenesis are still ambiguous. In fact, it has been hypothesized that NSG is a variant of sarcoidosis. Furthermore, infectious and hypersensitivity processes have been suggested to elicit the phenomenon [7]. NSG is sporadically confused with Wegener's granulomatosis, sarcoidosis, and Churg–Strauss syndrome. Evidently, Wegener's granulomatosis is a disease with upper respiratory tract symptoms, multifocal lung involvement, and renal manifestations with elevated anti-neutrophil cytoplasmic antibodies (ANCA).The histologic feature consists of suppurative granulomas with messy necrosis and necrotizing vasculitis which assist in distinguishing Wegener's granulomatosis from NSG. Another way of differential diagnosis is sarcoidosis which is characterized by well-formed nonnecrotizing granulomas. Apparently, necrotizing granulomas and necrotizing vasculitis with prominent eosinophils have been seen in Churg–Strauss syndrome.

In conclusion, it was magnificently attained that NSG is a disease with beneficial prognosis which should be considered as one of the differential diagnosis tools to identify necrotizing granulomatous diseases indeed.
